# Quantification of hazard prediction ability at hazard prediction training (Kiken-Yochi Training: KYT) by free-response receiver-operating characteristic (FROC) analysis

**DOI:** 10.1007/s12194-016-0374-1

**Published:** 2016-10-27

**Authors:** Masahiro Hashida, Ryousuke Kamezaki, Makoto Goto, Junji Shiraishi

**Affiliations:** 1 Department of Radiology, Hospital Division of Medical Technology, Kumamoto University Hospital, 1-1-1, Honjo, Chuo-ku, Kumamoto City, Kumamoto 860-8556 Japan; 2 Graduate School of Health Sciences, Kumamoto University, Kumamoto City, Kumamoto 860-8556 Japan; 3 Faculty of Life Sciences, Kumamoto University, Kumamoto City, Kumamoto 860-8556 Japan

**Keywords:** Kiken-Yochi Training (KYT), Free-response receiver-operating characteristic (FROC), Hazard prediction ability, Patient safety, Radiological technologist

## Abstract

The ability to predict hazards in possible situations in a general X-ray examination room created for Kiken-Yochi training (KYT) is quantified by use of free-response receiver-operating characteristics (FROC) analysis for determining whether the total number of years of clinical experience, involvement in general X-ray examinations, occupation, and training each have an impact on the hazard prediction ability. Twenty-three radiological technologists (RTs) (years of experience: 2–28), four nurses (years of experience: 15–19), and six RT students observed 53 scenes of KYT: 26 scenes with hazardous points (hazardous points are those that might cause injury to patients) and 27 scenes without points. Based on the results of these observations, we calculated the alternative free-response receiver-operating characteristic (AFROC) curve and the figure of merit (FOM) to quantify the hazard prediction ability. The results showed that the total number of years of clinical experience did not have any impact on hazard prediction ability, whereas recent experience with general X-ray examinations greatly influenced this ability. In addition, the hazard prediction ability varied depending on the occupations of the observers while they were observing the same scenes in KYT. The hazard prediction ability of the radiologic technology students was improved after they had undergone patient safety training. This proposed method with FROC observer study enabled the quantification and evaluation of the hazard prediction capability, and the application of this approach to clinical practice may help to ensure the safety of examinations and treatment in the radiology department.

## Introduction

Currently, various clinical safety measures are being implemented in many medical facilities to ensure patients’ safety. One of these measures is the introduction of Kiken-Yochi training (KYT) into clinical practice [1]. KYT is a hazard prevention technique (consisting of ensuring safety) in the workplace. It was invented in 1974 in Japan for prevention of occupational accidents, and, since the late 1970s, has spread from the manufacturing industry to the entire industrial world [2]. It is now being applied to various parts of society as an educational tool for the prevention of traffic accidents or as a leadership-training tool for dealing with children’s activities (e.g., Boy Scouts, a school trip) [3–5]. It has recently been introduced into clinical practice, and many books on its use in nursing have been published [6, 7].

In general, KYT can be defined as a particular training method, in which staff members first observe photos or illustrations of everyday scenes in a workplace, and then those scenes are discussed within a group for detection of potential hazards; countermeasures against hazardous issues are discussed, and, finally, slogans or messages are created directed at risk avoidance (ensuring of safety) [8, 9].

KYT as a safety measure (that is, as a hazard prevention technique) in clinical practice has been actively introduced into nursing and pharmaceutical management, and its efficacy in these areas has been reported [10, 11]. However, although KYT has also been introduced into the field of radiology, no adequate study on its efficacy has yet been reported. Yasuda et al. provided KYT to radiological technologists (hereafter referred to as RTs), and the results of a survey conducted before and after KYT revealed that KYT statistically significantly motivated a willingness to implement clinical safety. On the other hand, it also indicated that KYT had limitations with regard to events that were difficult to visualize (due to psychological factors) [12].

In KYT, it is important to be able to predict hazard points. Hazard points are that might cause injury to patients or potential hazards while scenes (photos or illustrations) are observed for the first time, as trainees cannot proceed on to the next stage of training without this prediction ability. In other words, hazard prediction ability at the first sight of a scene is connected directly to the ability to prevent a hazard, and high prediction ability is required for safety in clinical practice. The hazard prediction ability varies among individuals and may be affected by the years of clinical experience or by the individual’s occupation. There has thus far been no report on the measurement and qualification of hazard prediction ability.

In radiology, receiver-operating characteristics (ROC) analysis has been used for performing observer studies for evaluation of diagnostic accuracy or lesion detectability [13], and localization ROC (LROC) analysis for considering the location of a lesion has been performed in a computer-aided diagnosis (CAD) study [14]. Free-response ROC (FROC) analysis is used when a clinical situation needs to be reproduced (e.g., when there are multiple lesions) or when the performance of a CAD system is being assessed (e.g., concerning the number of false positives (FPs) per image) [15]. Generally, the diagnostic performances of two different systems (e.g., with and without CAD) can be evaluated by the same observer group in FROC analysis. However, a diagnostic performance of individual observer in the lesion (abnormality) detectability can be compared by use of FROC analysis [13, 15]. Application of this FROC analytical approach to the viewing of scenes in KYT may allow an evaluation of the ability of an observer to identify hazard points or their degree (the likelihood of hazards) and to quantify the hazard prediction ability.

In this study, we aimed to quantify the hazard prediction abilities of RTs viewing scenes in KYT by using FROC analysis and investigated the effects of the total number of years of clinical experience or experience in working on general X-ray examinations with regard to their hazard prediction ability. In addition, differences in occupations of nurses and the improvement of hazard prediction ability among RT students who have the national license of a radiological technologist were investigated.

## Methods

### KYT scenes used in FROC study

Scenes of simulated situations before a general X-ray examination (hereafter referred to simply as examination), during examination, after examination in an X-ray room and the movement of the patient, were all pictured with a digital camera, simulating one patient and one to two RTs. To simulate the KYT scenes used in the FROC study, we took into account forty reports on incidents which had occurred in the X-ray room of the radiology section of our hospital over the past 10 years [16]. These incident reports included patients falling and tumbling (29.2%), incorrect identification of patient (25.0%), incorrect positioning (12.5%), patient injury (except from falling and tumbling) (10.4%), a malfunctioning device (10.4%), accidental removal of patient’s tubes (4.2%), and other events (8.4%), in descending order of frequency. Simulating ‘incorrect identification of patient’, ‘incorrect positioning’, and ‘malfunctioning device’ was difficult; therefore, scenes of ‘patient falling and tumbling’, ‘patient injury (finger getting caught in the table, bruise, etc.)’, and ‘accidental removal of patient’s tubes’ were created as hazard points. In addition to scenes with hazard points, scenes without hazard points were also created.

Thirty-five scenes with hazard points and forty scenes without hazard points were created, and these scenes were observed by three individuals: a technologist in charge of the general X-ray examination section at our department (15 years of experience out of 24 years of total clinical experience), a technologist at another hospital (15 years of experience out of 35 years of total clinical experience), and a teaching staff member of a radiology department at another university (10 years of experience out of 28 years of clinical experience).

**Fig. 1:**
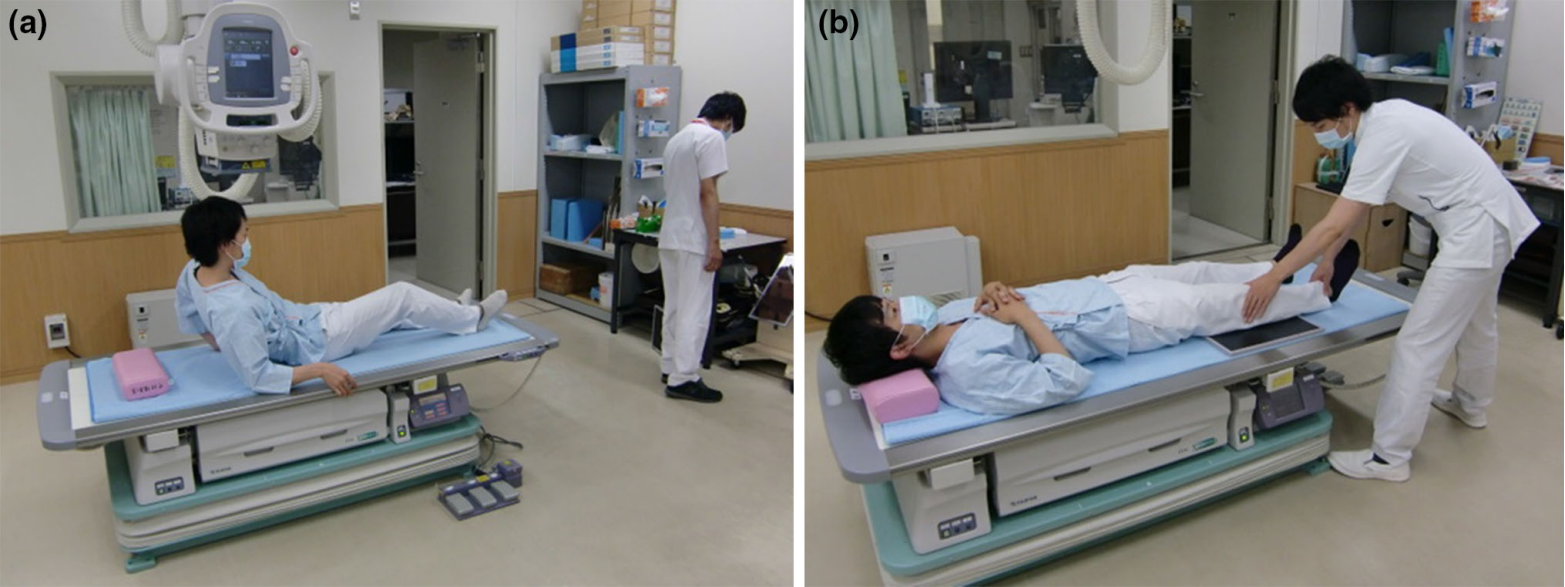
Examples of scenes in this observer study. **a** A scene with hazard points. **b** A scene with no hazard point

**Fig. 2:**
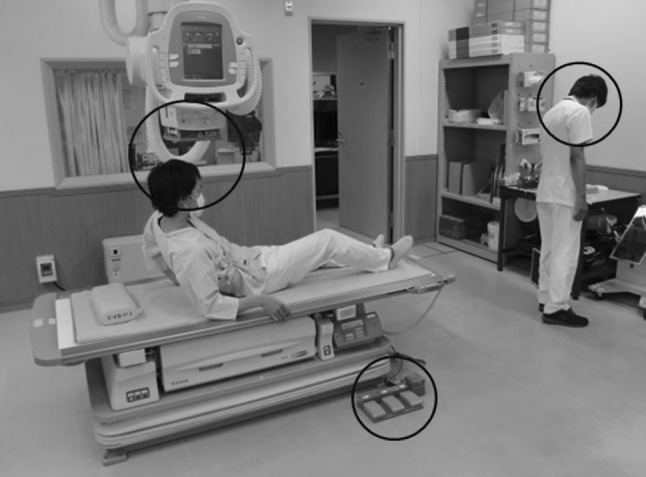
An example of a scene showing hazard areas (*red circle*). A hazard area is the same as a TP area in an analysis of FROC

After observation, scenes with and without hazard points were determined through consensus of the three individuals. As a result, 53 scenes in total (26 scenes with hazard points and 27 scenes without hazard points) were selected as a sample set to be used in an observer study. Examples of scenes with and without hazard points are shown in Fig. 1a and b, respectively. Moreover, the above-mentioned three individuals agreed to define the center locations and the range of hazard points for the 26 scenes with hazard points, and an observer’s response was determined as true positive (TP) when a mark of his/her response was located within the range of a hazard point. The observer provided a point for the hazard point by clicking a mouse. For example, when an observer indicated a mark within a range including a patient’s head and a multiple collimator unit on an X-ray tube as the range of hazard points in an event in which a patient hit his/her head on an X-ray tube, all indications in this range were handled as a TP. However, when multiple TPs were found in the range of the same hazard point, the only most likely TP was employed and the other TPs were excluded from the calculation. When an observer indicated a mark within a range other than that specified as a hazard point, all responses were determined as FP. Figure 2 shows scenes indicating the range of hazard points. The range of a hazard point was always a circle.

In scenes with hazard points, one to three hazard points causing events were set per scene, creating a total of 42 points. Events estimated from hazard scenes included ten of tumbling (14 hazard points), five of falling, five of patient injury (seven hazard points), four of a finger getting caught in the table, four of accidental removal of patient’s tubes, and two of device damage. The total number of events was greater than the number of scenes, because multiple events could occur in a single scene. Furthermore, six points, including scenes in which RTs were looking away or were away from a patient, were included as hazard points. Scenes of different regions of examinations included four heads, nine chests, eleven abdomens, three pelvises, nine upper extremities, eleven lower extremities examinations, and six scenes of patient movement.

### Observation experiment

Twenty-three RTs with different numbers of years of clinical experience were included as observers and were divided into five groups: those with clinical experience of less than 5 years (*n* = 5), 5 to less than 10 years (*n* = 6), 10 to less than 15 years (*n* = 4), 15 to less than 20 years (*n* = 4), and more than 20 years (*n* = 4). The 11 RTs with clinical experience of less than 10 years were further divided into two groups: those with examination experience in the past 2 years (*n* = 5) and those with no experience (*n* = 6). Of the 12 RTs with clinical experience of 10 years or more, none had had examination experience in the past 2 years. In addition, we included four nurses (with 15 to 19 years of clinical experience) and six RT students in this observer study. Note that all RT students already had a national license of RT and worked as RTs one to two times a week at hospitals.

For investigation of the effect of educational training related to KYT, the six RT students performed the same observer study twice, before and after having educational training. We used a publicly available DVD [17] on patient safety education for their training. A 5-month interval was set between their two observer studies (i.e., before and after training). The details of this experiment were explained to the observers, and written consent was obtained from all observers.

ROC Viewer, a software for ROC/FROC observer study developed by Shiraishi et al., was used in this FROC observer study [18]. Observers were asked to view 53 randomly presented scenes one by one, and to indicate hazard points by mouse clicking. When clicking the hazard points, the observers entered the likelihood of a hazard (0.01–1.0), ranging from “possibly hazardous” to “definitely hazardous” on a continuous rating scale. There was no limitation on viewing distance and observation time, and the observers were allowed to take a break during the observation. Observers were notified about the definition of hazard points; hazard points were situations that might cause injury to patients, but not to RTs. In typical KYT, observers view a scene and predict hazardous conditions while estimating the next scene. However, in this study, observers were asked to indicate hazard points in a scene without proceeding to the next scene, because their hazard prediction abilities at the first viewing of a scene were being analyzed.

Although a statistical comparison of average FROC curves obtained from two different systems can be made by use of the analysis software Jackknife Alternative FROC (JAFROC) [19], JAFROC software does not correspond to the creation of a single AFROC curve [20], and the calculation of the figure of merit (FOM) [19], which can be considered as the area under the ROC curve, of each observer. Therefore, our original software, which was created based on the theory described in Ref. [19], was used for creation of an AFROC curve from the observation results, and the FOM of each observer was simultaneously calculated. Statistically significant differences (significance level: 5%) in the means of the hazard prediction abilities in each groups were confirmed by use of a *t* test based on the FOM of each observer with StatView, a software for statistical analysis.

## Results

**Fig. 3:**
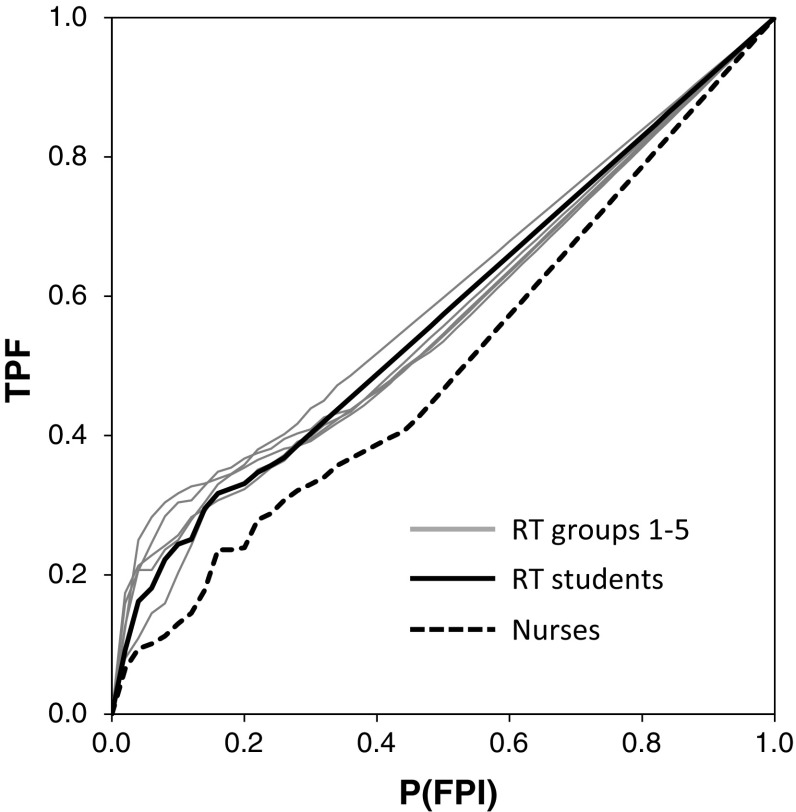
Average AFROC curves for the recognition of KYT, obtained from five groups of radiological technologists (RTs), one group of RT students, and one group of nurses

Table 1 shows the average of the FOM, sensitivity, and specificity for the five RT groups, six RT students, and four nurses. Figure 3 shows the average AFROC curve for each group. The average FOM was the lowest for the nurses, followed by RTs with 5–10 years of clinical experience, RT students, RTs with 15–20 years of clinical experience, RTs with 10–15 years of clinical experience, RTs with 5 years or less of clinical experience, and RTs with 20 years or more of clinical experience. No significant differences between RT groups and between RTs and RT students were observed in the test results, whereas significant differences were found between nurses and RTs with 20 years or longer clinical experience and between nurses and RT students.

**Table 1:**
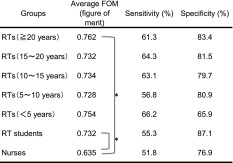
Average figure of merit (FOM), sensitivity and specificity obtained from FROC observer study for evaluating the ability of KYT, performed by five groups of radiological technologists (RTs), one group of RT students, and one group of nurses

*RTs* radiological technologists (the years of clinical experience)

* Statistically significant difference *p* < 0.05

**Fig. 4:**
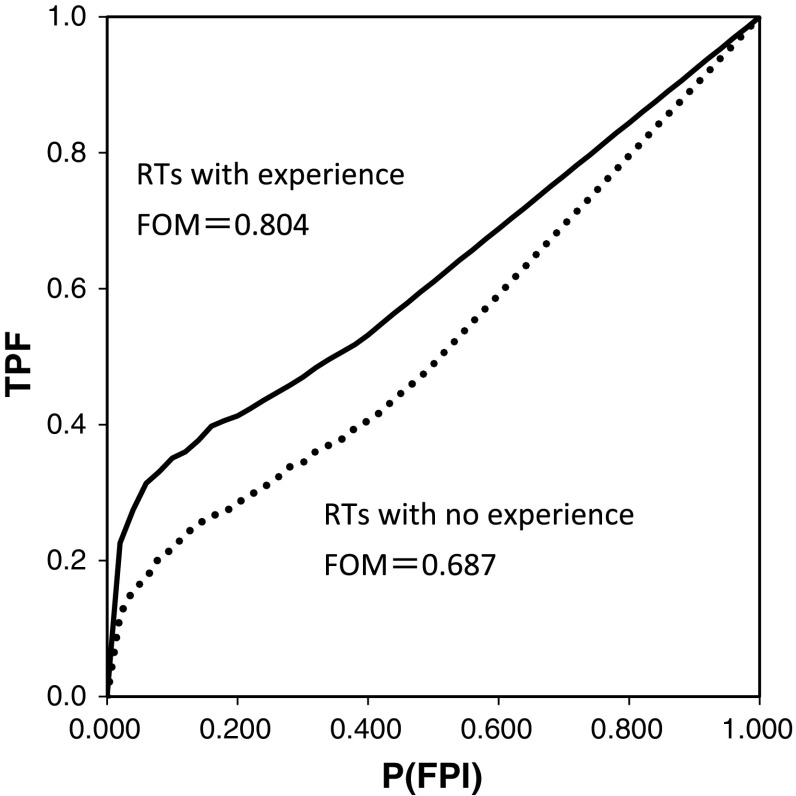
AFROC curves for the recognition of KYT, obtained from two groups of radiological technologists (RTs) with (*solid*) and without (*dash*) experience in general X-ray examinations within 2 years. Note that all RTs had less than 10 years’ experience in clinical work

Figure 4 shows the average AFROC curves for the eleven RTs with less than 10 years of clinical experience, divided into RTs with examination experience in the past 2 years (*n* = 5) and RTs without such experience (*n* = 6). The average FOMs were 0.804 and 0.687 for RTs with and without experience, respectively, indicating a significant difference (*p* = 0.018).

The average FOM for RT students was higher after they had watched the patient safety training DVD (0.757) than it was before watching (0.732), as shown in Table 2. However, no significant difference was observed (*p* = 0.295).

**Table 2:** Figure of merit (FOM), sensitivity, and specificity obtained from 6 RT students by use of FROC observer study before and after having the educational training for KYT

RT students	FOM (figure of merit)			Sensitivity (%)		Specificity (%)	
	Before	After	ΔFOM	Before	After	Before	After
S1	0.713	0.799	0.086	45.2	54.8	100.0	100.0
S2	0.729	0.788	0.059	57.1	66.7	81.5	88.9
S3	0.743	0.764	0.021	57.1	78.6	85.2	66.7
S4	0.707	0.751	0.044	47.6	59.5	85.2	88.9
S5	0.746	0.756	0.010	57.1	61.9	81.5	88.9
S6	0.751	0.683	−0.068	66.7	61.9	88.9	51.9
Average (S1–S6)	0.732	0.757	0.025	55.1	63.9	87.1	80.9
Average (S1–S5)	0.728	0.772	0.044*	52.8	64.3	86.7	86.7

Average FOMs were obtained for two groups of (S1–S6) and (S1–S5)

* Statistically significant difference *p* = 0.031

## Discussion

Sensitivity to clinical safety hazards and ability to predict hazard points (situations) by individual medical staff members in a hospital are essential for implementing reliable diagnoses and treatments that ensure patients’ safety. In this study, we applied FROC analysis to scene observation in KYT to quantify the hazard prediction ability of RTs, RT students, and nurses.

No significant differences between the numbers of years of clinical experience and between the FOMs for RTs were observed (Table 1). However, in an analysis of eleven RTs with less than 10 years of clinical experience divided into 2 groups—those with and those without examination experience in the past 2 years—, the FOM was significantly higher in the group with experience (Fig. 4). Generally, the hazard prediction ability is expected to increase with clinical experience. Takeuchi et al. reported that the risk of incidence of irregular procedures would increase for those with less work experience in physical therapy and occupational therapy departments, based on an analysis of previous incidents [21]. According to a survey of sensitivity of RTs to patient safety conducted by Doi et al., RTs with less than 6 years of clinical experience considered their own hazard prediction abilities to be low, whereas RTs with experience of 7 years or longer considered their own hazard prediction abilities to be intermediate [22]. However, no statistically significant difference between the total years of clinical experience and the hazard prediction ability (FOM) was observed in our study.

According to the results of an analysis of incident reports over the past 10 years conducted by Hashida and Shiraishi [16], RTs with experience of 6 years or longer accounted for more than half (55%) of the reports of incidents, and this result does not conflict with the results of this study. A significant difference in the FOM was found in our study between RTs with and those without examination experience in the last 2 years. Hazard prediction abilities are likely to be higher for RTs currently engaged in examination work or RTs who have fresh experience with examinations. The reason that the FOM was small for nurses may be that they are not familiar with the details of an X-ray examination. In other words, knowledge of clinical practice and recent examination experience may have a greater impact on hazard prediction ability than total years of clinical experience.

There was no significant difference between RT students and any of the RT groups (Table 1). However, a significant difference was observed between the average FOM (0.732) of RT students before educational training and that (0.804) of the six RTs with examination experience in the last 2 years (*p* = 0.008). RT students have some experience (and thus are not new to work in radiology), even though they have once or twice-a-week working experience in hospitals. Therefore, the hazard prediction ability of RT students may not have reached the level of RTs who are currently engaged in examination work. RT students may have hazard prediction abilities comparable to those of RTs who had examination experience only in the remote past. This also may be due to the fact that actual experience in clinical practice has an impact on hazard prediction.

No significant difference for the RT students was observed in the FOMs before and after patient safety training through watching a DVD. However, a significant difference was detected in the FOMs before and after patient safety training when one RT student (S6 in Table 2) was excluded (*p* = 0.031). An analysis of the observation results for this RT student showed that the specificity was drastically reduced in the observations made after the training (88.9 to 51.9%) (Table 2), indicating a pronounced tendency to be “too cautious (over reading)”. Being too cautious (detecting a larger number of hazard points) is a favorable tendency in terms of patient safety. Therefore, patient safety training in which observers are asked to watch a DVD may improve the hazard prediction ability. Hence, this approach is suggested to be useful for evaluation of hazard prediction ability.

In this study, sensitivity was defined as the percentage of “hazard points” that an observer correctly identified among 42 hazard points, and specificity was defined as the percentage of scenes that an observer correctly identified as “without hazard points” among 27 scenes. Because the sensitivity was less than 70% for almost all observers, the observers had a tendency to overlook hazard points. According to the results of our analysis of individual observers’ data, it was inferred that hazard point, when “RTs were looking away or were away from a patient”, had the highest frequency in cases where a hazard point(s) was overlooked, and the result on this item was dependent on the judgment criteria of the observing individual. Moreover, the above sensitivity was a result of calculating a percentage of the 42 hazard points, but when the percentage was re-calculated on 30 cases (events caused by hazard points), the sensitivity was 56.3–90.6%. The re-calculated sensitivity was higher than the original sensitivity for all observers. It was concluded that an observer had a tendency to overlook other hazard points in a scene when the observer identified one hazard point in the same scene.

Questionnaires targeting students or staff in a hospital are frequently used in studies of clinical safety [10–12, 22, 23] and enable to indirectly assess sensitivity and behavior regarding safety. In this study, the observers’ activities (abilities) were directly measured and quantified by use of an FOM based on the hazard points indicated by observers. Quantification of safety and hazards is important in the assessment of countermeasures for clinical safety and prevention of hazards; thus, it will be essential for promoting clinical safety.

In this FROC analysis, a TP was defined when observers indicated hazard points created by the consensus of three expert RTs, whereas an FP was defined when observers indicated other points and/or scenes without hazard points. Even though these FPs were not ultimate hazard points, being too cautious (indicating FP) means that observers may have perceived a potential hazard at their own discretion. Therefore, in the future, we need to examine hazard prediction ability while considering FPs, as well as the FOM.

Although some scenes with patient movement can be shared with other departments, the results of this study are limited to hazards predicted by RTs in an X-ray examination room. Hazard prediction abilities determined in this study were based on the safety rules of our hospital; detailed guidelines for clinical safety vary among hospitals. In addition, the evaluation of hazard prediction abilities may vary depending on the scenes used in KYT. It is therefore desirable to divide scenes into those common among departments or hospitals (patient movements etc.,) and those specific to a certain department or hospital (in an X-ray room, for example), for future investigations on the relationship between experience in clinical practice and hazard prediction ability.

## Conclusion

The hazard prediction abilities of RTs, RT students, and nurses regarding scenes in KYT in an X-ray examination room were quantified by the use of FROC analysis, for investigating whether the number of years of experience, the occupation, and the efficacy of training influenced hazard prediction ability. The results showed that the total number of years of clinical experience had no effect on the hazard prediction ability; however, recent X-ray examination experience had a greater impact. Differences in the observers’ occupations caused differences in their hazard prediction abilities even in observing the same scenes, and the hazard prediction ability of RT students was improved after patient safety training. Using this proposed method using the FROC observer study enabled quantification and evaluation of hazard prediction ability. Therefore, the introduction of this approach into clinical practice may help ensure patient safety in radiology departments.
